# Does animacy affect visual statistical learning? Revisiting the effects of selective attention and animacy on visual statistical learning

**DOI:** 10.1177/17470218231173883

**Published:** 2023-06-02

**Authors:** Jolene A Cox, Yizhou Wu, Anne M Aimola Davies

**Affiliations:** 1 School of Medicine and Psychology, The Australian National University, Canberra, ACT, Australia; 2 Centre for Human Factors and Sociotechnical Systems, University of the Sunshine Coast, Maroochydore, QLD, Australia

**Keywords:** Animate-monitoring hypothesis, conscious awareness, implicit learning, selective attention, visual statistical learning

## Abstract

Animates receive preferential attentional processing over inanimates because, from an evolutionary perspective, animates are important to human survival. We investigated whether animacy affects visual statistical learning—the detection and extraction of regularities in visual information from our rich, dynamic, and complex environment. Participants completed a selective-attention task, in which regularities were embedded in two visual streams, an attended and an unattended visual stream. The attended visual stream always consisted of line-drawings of non-objects, while the unattended visual stream consisted of line-drawings of either animates or inanimates. Participants then completed a triplet-discrimination task, which assessed their ability to extract regularities from the attended and unattended visual streams. We also assessed participants’ awareness of regularities in the visual statistical learning task, and asked if any learning strategies were used. We were specifically interested in whether the animacy status of line-drawings in the unattended visual stream would affect visual statistical learning. There were four key findings. First, selective attention modulates visual statistical learning, with greater visual statistical learning for attended than for unattended information. Second, animacy does not affect visual statistical learning, with no differences found in visual statistical learning performance between the animate and inanimate condition. Third, awareness of regularities was associated with visual statistical learning of attended information. Fourth, participants used strategies (e.g., naming or labelling stimuli) during the visual statistical learning task. Further research is required to understand whether visual statistical learning is one of the adaptive functions that evolved from ancestral environments.

At any given moment, our visual system is faced with more sensory information than it is capable of processing. Visual attention acts to filter the available sensory information, by selecting information important to, and relevant for, adaptive behaviour for further processing (Desimone & Duncan, 1995). Evolutionary psychologists (e.g., Cosmides & Tooby, 2013) have suggested that information about animates (e.g., living things, such as a lion or a baby) is both important and relevant for adaptive behaviour, which is why animates receive preferential attentional processing over inanimates (e.g., non-living things, such as a spoon or a cup).

From birth, infants have a fundamental ability to distinguish animates from inanimates (e.g., Klein & Jennings, 1979; Legerstee et al., 1987) and this ability forms the basis of their social and cognitive development (Legerstee, 1992). Over time, this ability develops further, as interactions with animates and inanimates increase (see review by Gelman & Opfer, 2002). Although animates are broadly referred to as living things and inanimates as non-living things, there are four key distinctions between the two animacy categories (Bonin et al., 2014; but see Gelman & Spelke, 1981). According to Bonin et al.,

(1) animates can act, whereas inanimates move only when something/someone initiates the action; (2) animates grow and reproduce; (3) animates can know, perceive, emote, learn, and think; and (4) animates are made of biological structures that maintain life and allow reproduction. (p. 370)

New et al. (2007) suggested that visual attention selects information based on “category-specific” criteria, including a criterion that differentially monitors animates (“the animate-monitoring hypothesis”). They explained that this criterion meant that animates would receive preferential processing over inanimates, even when animates are unexpected or irrelevant to the observer’s current goals or activities. The preferential processing of animates over inanimates represents a consequence of evolutionary-adapted mechanisms that were developed to ensure the survival of our ancestors. For ancestral hunter–gatherers immersed in rich biotic environments, animates would have been regarded as more important and thus they required responses that were more time-sensitive because animates convey signs of social opportunities, or threat and danger.

The animate-monitoring hypothesis has been tested in several visual perception and cognition tasks over the last two decades (e.g., Altman et al., 2016; Calvillo & Hawkins, 2016; Guerrero & Calvillo, 2016; Jackson & Calvillo, 2013; New et al., 2007). For example, Altman et al. (2016) and New et al. (2007) tested the animate-monitoring hypothesis using the change blindness task. New et al. reported that participants were faster and more accurate at detecting the changed object (reduced change blindness) when the object belonged to an animate category (animate-change object) than when the object belonged to an inanimate category (inanimate-change object). Altman et al. reported that participants were faster and more accurate at detecting the inanimate-change object when a distractor that belonged to an animate category was positioned near to (i.e., in close physical proximity), compared with when it was positioned far from (i.e., in distant physical proximity), the inanimate-change object. Calvillo and Hawkins (2016) and Calvillo and Jackson (2014) also tested the animate-monitoring hypothesis using the inattentional blindness task. They reported that participants demonstrated greater detection of the unexpected stimulus (reduced inattentional blindness) when the stimulus was from the animate category than when the stimulus was from the inanimate category. The animate-monitoring hypothesis was also tested in other tasks, such as the Stroop task (Bugaiska et al., 2019), the attentional blink task (Guerrero & Calvillo, 2016), and the visual search task (Jackson & Calvillo, 2013). Evidence from across these studies provided support for the animate-monitoring hypothesis—the hypothesis that animates receive preferential attentional processing over inanimates (“animacy advantage”).

While many studies provided support for the animate-monitoring hypothesis, a few studies did not find support for this hypothesis as their findings suggest no advantage or influence of animacy on task performance (e.g., Cox et al., 2022; Hagen et al., 2018; Hagen & Laeng, 2016, 2017). Of most relevance to the present study is a previous study by our laboratory group that examined whether animacy affects visual statistical learning of temporal regularities—the ability to extract regularities in how objects within our visual environment appear in relation to each other across time (Cox et al., 2022). More specifically, the study examined the effects of selective attention and animacy on visual statistical learning of temporal regularities, by measuring and comparing visual statistical learning of attended and unattended information, across four animacy conditions. The four animacy conditions tested were: (1) living things that can self-initiate movement (i.e., animals), (2) living things that cannot self-initiate movement (i.e., fruits and vegetables), (3) non-living things that can generate movement (i.e., vehicles), and (4) non-living things that cannot generate movement (i.e., tools and kitchen utensils). Visual statistical learning was measured using a task that involved the completion of two phases—a familiarisation phase and a test phase. In the familiarisation phase, participants were exposed to a continuous visual stream containing green line-drawing stimuli and red line-drawing stimuli from one of the four animacy conditions tested. Participants were asked to attend either to the green or to the red stimuli (“attended visual stream”) and were asked to make a keyboard response when they detected that the stimulus in the attended visual stream was identical to the last stimulus they observed in that same visual stream (i.e., a stimulus repetition). This task was a selective-attention task, which was intended to bias participants’ attention towards one of the two visual streams. Participants were not aware that in each visual stream (i.e., attended visual stream and unattended visual stream), there were regularities embedded; that is, the line-drawings were grouped into triplets. Following the completion of the familiarisation phase, participants completed the test phase, which involved a two-interval forced-choice (2IFC) triplet-discrimination task. On each trial of the triplet-discrimination task, participants were first presented with two triplets sequentially, and then asked to indicate which one of the two triplets presented was more familiar based on the information from the familiarisation phase. One of the triplets was a triplet that appeared in the familiarisation phase (“familiar triplet” from either the attended or unattended visual stream) and the other triplet never appeared in the familiarisation phase (“impossible triplet”). Performance on the triplet-discrimination task was used as a measure of visual statistical learning.

In the Cox et al. (2022) study, we reported that visual statistical learning was modulated by selective attention, with greater visual statistical learning for attended information than for unattended information. Participants demonstrated better performance for attended-triplet discrimination than for unattended-triplet discrimination. However, animacy did not affect visual statistical learning. Visual statistical learning was observed in all four animacy conditions (living things that can self-initiate movement, living things that cannot self-initiate movement, non-living things that can generate movement, and non-living things that cannot generate movement) but visual statistical learning did not differ across these animacy conditions. The finding—that animacy did not affect visual statistical learning—could be attributed to the task design (Cox et al., 2022). Of note is that the stimuli in the attended and unattended visual stream for each animacy condition belonged to the same category. For example, for the animacy condition “living things that can self-initiate movement”, participants were presented with line-drawings of animals in both the attended and unattended visual streams. This task design may have attenuated or eliminated the effects of animacy. A more appropriate task design would involve manipulating the animacy status of stimuli in the unattended visual stream only, and then testing whether this animacy manipulation would affect visual statistical learning of attended and unattended information. We consider this task design to be more appropriate for measuring the effects of animacy because previous studies have investigated the effects of animacy on visual perception and cognition by manipulating the animacy status of unattended stimuli (e.g., Altman et al., 2016; Calvillo & Jackson, 2014). These studies have found that, even when the animacy status of stimuli were task-irrelevant and were intended to be ignored by the observer, animacy did affect task performance. This is because the core premise behind the animate-monitoring hypothesis is that animacy is a privileged category in perceptual processing.

The present study extends our previous investigation (Cox et al., 2022) of whether animacy affects visual statistical learning of attended and unattended information. In the present study, we manipulated the animacy status of the unattended information in the visual statistical learning task, such that, participants were presented with line-drawings of either living things that can self-initiate movement (unattend-animate; i.e., animals) or non-living things that cannot generate movement (unattend-inanimate; i.e., tools and kitchen utensils) in the unattended visual stream of the familiarisation phase. Participants were always presented with line-drawings of non-objects in the attended visual stream of the familiarisation phase.

Three predictions were made. First, based on previous studies demonstrating reliable effects of selective attention on visual statistical learning, we predicted overall greater visual statistical learning for attended information compared with visual statistical learning for unattended information. Second, based on the animate-monitoring hypothesis, we predicted that visual statistical learning of attended information would differ across the two animacy conditions (unattend-animate, unattend-inanimate). More specifically, we predicted poorer visual statistical learning for attended information by participants in the unattend-animate condition compared with participants in the unattend-inanimate condition. This is because, compared with the presence of line-drawings of inanimates in the unattended visual stream, the presence of line-drawings of animates in the unattended visual stream would capture participants’ attention, resulting in poorer visual statistical learning for attended information. Third, we also predicted that visual statistical learning of unattended information would differ across the two animacy conditions (unattend-animate and unattend-inanimate). More specifically, we predicted greater visual statistical learning for unattended information by participants in the unattend-animate condition compared with participants in the unattend-inanimate condition. This is because, when compared with the presence of line-drawings of inanimates in the unattended visual stream, the presence of line-drawings of animates in the unattended visual stream would capture participants’ attention, resulting in greater visual statistical learning for unattended information. Support for either the second or third prediction would provide evidence for the animate-monitoring hypothesis, by demonstrating that animacy affects visual statistical learning.

In addition to investigating whether animacy affects visual statistical learning, we examined whether participants were aware of the regularities in the visual statistical learning task by administering an assessment of awareness (4-point confidence-rating scale) during the triplet-discrimination task. Participants’ knowledge of the regularities in the visual statistical learning task is considered to be under the subjective threshold of consciousness if the following two criteria are met: the guessing criterion and the zero-correlation criterion (Cheesman & Merikle, 2001; Dienes, 2004). The guessing criterion is met if participants demonstrate above-chance performance on the triplet-discrimination task despite reporting being not confident (or guessing) of their responses for triplet discrimination. The zero-correlation criterion is met if participants’ confidence of their responses for triplet discrimination is not correlated with their performance on the triplet-discrimination task. The administration of an assessment of awareness has not been adopted widely in visual statistical learning studies, but we have previously administered an assessment of awareness (4-point confidence-rating scale) in our studies (Cox & Aimola Davies, 2022; Cox et al., 2022). An approach commonly adopted in the existing literature is to ask participants to self-report, at the end of the visual statistical learning task, whether they noticed any regularities during the familiarisation phase (e.g., Otsuka et al., 2013; Turk-Browne et al., 2005). In line with these studies, we also included this self-report measure at the end of our triplet-discrimination task as part of a Post-Experiment Questionnaire. In addition, in this questionnaire, we examined whether participants named or labelled the stimuli during the familiarisation phase and whether participants adopted any strategies during the familiarisation phase, strategies that would help them complete the task.

## Method

### Participants

Undergraduate students at the Australian National University (ANU) were invited to take part in the present study. Eligible participants had to be aged between 18 and 30 years, right-handed, and have no history of cognitive or neurological impairments. Participants received monetary compensation (AUD15) or, if eligible, received course credits for their participation (one course credit). Ethical approval for this study was obtained from the ANU Human Research Ethics Committee (Protocol 2019/795).

A total of 57 young neurologically healthy adults participated in the present study. Five participants were excluded before data analyses due to poor performance on the selective-attention task in the familiarisation phase (proportion of hits below 0.70 and number of false alarms above 20). The exclusion criteria were established prior to data collection. The final sample consisted of 52 participants, of which 23 were male and 29 were female. The mean age of the sample was 21.75 years (*SD* = 2.89, range = 18–29 years). Twenty-six participants completed the visual statistical learning task with line-drawings of animates in the unattended stream (unattend-animate) and the remaining 26 participants completed the visual statistical learning task with line-drawings of inanimates in the unattended stream (unattend-inanimate).

### Apparatus

The visual statistical learning task was programmed using Inquisit 6 (Millisecond Software, WA, USA) and displayed on a Dell Liquid-Crystal Display computer with a refresh rate of 60 Hz and a screen resolution of 1,920 by 1,080 pixels. Participants’ eye-to-screen viewing distance was estimated at 57 cm. A Dell keyboard was used to record participants’ responses.

### Visual statistical learning task

There were two phases in the visual statistical learning task: (1) the familiarisation phase and (2) the test phase. In the familiarisation phase, participants were presented with one single, combined visual stream of green-coloured line-drawing stimuli and red-coloured line-drawing stimuli. The next subsection details the construction of each of the green- and red-coloured visual streams, and the integration of these two visual streams into a single, combined visual stream.

#### Construction of the single, combined visual stream

**Figure 1.**
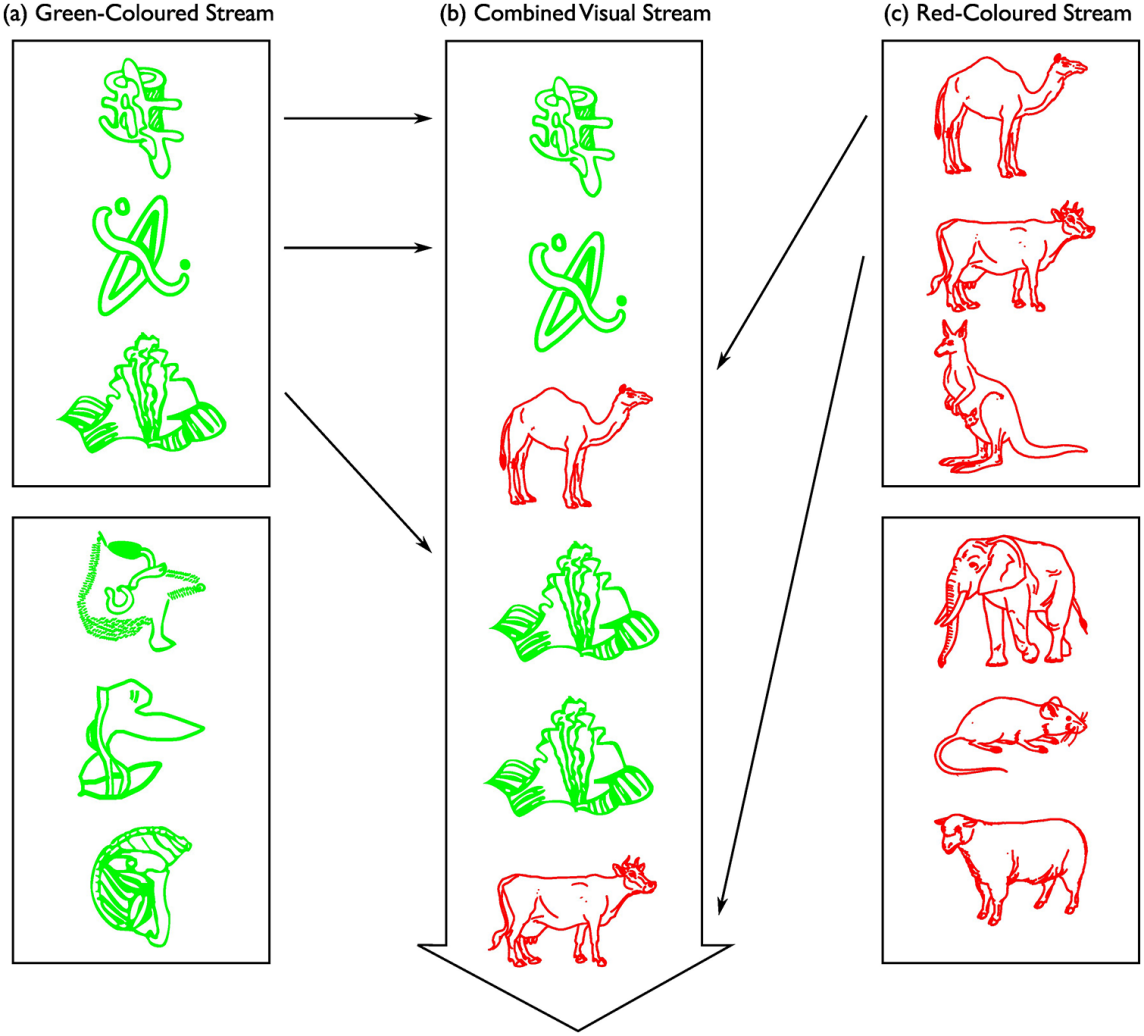
Construction of the single, combined visual stream in the familiarisation phase of the visual statistical learning task. There were two coloured visual streams in the familiarisation phase of the visual statistical learning task: (a) green-coloured visual stream and (c) red-coloured visual stream. Stimuli in each of the coloured visual streams were grouped into four triplets. The figure above illustrates the examples of two triplets in each coloured visual stream. After the construction of each of the coloured visual streams, the two streams were interleaved into (b), a single, combined visual stream. Participants were presented with this single, combined visual stream. Each stimulus appeared individually in the centre of the computer display. In this example, participants were asked to attend to the stimuli in the green-coloured stream (“attended-colour stream”), and to make a keyboard response when they detected a stimulus repetition (when the stimulus in the attended-colour stream was identical to the last stimulus in that same stream). In the figure, the stimulus repetition is the fourth green-coloured stimulus in (b), the single, combined visual stream.

Each of the two visual streams of green-coloured line-drawing stimuli and red-coloured line-drawing stimuli was constructed independently (see Figure 1a and c for an example of the two coloured visual streams). In each coloured visual stream (green or red), there were 12 stimuli (grouped into four triplets) repeated 24 times (= 288 stimuli). The third stimulus of each of the four triplets was repeated six times (= 24 stimuli) to generate the stimulus repetitions for the selective-attention task. In total, each coloured stream (green or red) consisted of 312 stimuli (= 288 stimuli and 24 stimulus repetitions). There were two constraints for the construction of each coloured visual stream: (1) no repeated triplets were allowed (i.e., . . . triplet *A–B–C*, triplet *A–B–C*, . . .) and (2) no repeated pairs of triplets were allowed (i.e., . . .triplet *A–B–C*, triplet *D–E–F*, triplet *A–B–C*, triplet *D–E–F*, . . .). These constraints applied, irrespective of whether a stimulus repetition occurred. For example, the sequence 'triplet *A–B–C*, followed by a stimulus repetition *C*, and then triplet *A–B–C’* never occurred in the construction of each coloured visual stream. The order of stimulus presentation in each triplet was fixed, such that, triplet *A–B–C* was always presented as triplet *A–B–C* and never presented as triplet *B–C–A* or *C–B–A*. The assignment of the stimuli within each triplet was randomised between participants. The joint probabilities of triplet (0.083 without stimulus repetitions and 0.077 with stimulus repetitions) and non-triplet (0.027 without stimulus repetitions and 0.019 with stimulus repetitions) combinations for each coloured visual stream were identical to the joint probabilities in the study by Turk-Browne et al. (2005).

After the construction of each of the coloured visual streams, the two streams were interleaved into a single, combined visual stream. In total, the single, combined visual stream consisted of 624 stimuli (= 312 stimuli from the green-coloured visual stream and 312 stimuli from the red-coloured visual stream). Figure 1b depicts an example of the single, combined visual stream. There was one constraint for the construction of the single, combined visual stream, which was that the number of stimuli from one coloured visual stream never exceeded the number of stimuli from the other coloured visual stream by more than six stimuli. The joint probabilities of triplet (0.01 without stimulus repetitions and 0.01 with stimulus repetitions) and non-triplet (0.003 without stimulus repetitions and 0.002 with stimulus repetitions) combinations in the single, combined visual stream were identical to the joint probabilities in the study by Turk-Browne et al. (2005).

### Stimuli

There were two different animacy conditions tested: (1) unattend-animate (i.e., animals) and (2) unattend-inanimate (i.e., tools and kitchen utensils), with 24 different line-drawings in each of the two categories. For each of the two animacy conditions, there were three identical sets of the 24 line-drawings, and these were coloured green and red in the familiarisation phase, and coloured black in the test phase. The line-drawings of animates and inanimates were obtained from the pictorial database by Snodgrass and Vanderwart (1980). Each line-drawing subtended a visual angle of approximately 10.25° by 5.78°. The line-drawings of non-objects were redrawn from the pictorial set by Kroll and Potter (1984).

The animacy status of the line-drawing stimuli was manipulated only in the unattended-colour stream and not in the attended-colour stream, such that, participants were presented with either line-drawings of animates in the unattended-colour stream or line-drawings of inanimates in the unattended-colour stream. Participants were always presented with line-drawings of non-objects in the attended-colour stream.

### Procedure

Participants provided written, informed consent at the beginning of the testing session. Participants were tested in groups, with a maximum of four participants in each group, in a distraction-free computer laboratory room. The study was described to participants as a visual processing task. Importantly, participants did not receive information about the triplet groupings (i.e., temporal regularities) in the familiarisation phase of the task, or information that they would be completing a triplet-discrimination task based on the information from the familiarisation phase. Participants completed a brief practice exercise for the selective-attention task in the familiarisation phase, but not for the triplet-discrimination task in the test phase.

#### Selective-attention task in the familiarisation phase

Participants were informed that they would be presented with a visual stream of green-coloured and red-coloured stimuli, with each stimulus appearing individually in the centre of the computer display. Half of the participants were instructed to attend to the green-coloured stimuli and the other half were instructed to attend to the red-coloured stimuli. For the participants who were instructed to attend to the green-coloured stimuli, the green-coloured stimuli were the attended-colour stream and the red-coloured stimuli were the unattended-colour stream. For the participants who were instructed to attend to the red-coloured stimuli, the red-coloured stimuli were the attended-colour stream and the green-coloured stimuli were the unattended-colour stream. Each stimulus was presented for 800 ms, followed by an interstimulus duration of 400 ms (i.e., a stimulus-onset asynchrony of 1,200 ms).

While monitoring the attended-colour stream, participants were asked to make a keyboard response when they detected a stimulus repetition in the attended-colour stream; that is, when a stimulus was identical to the last stimulus they observed in that same stream. The stimulus repetition in the attended-colour stream could be interrupted by the presentation of other stimuli in the unattended-colour stream. In total, there were 24 stimulus repetitions requiring participants’ responses. This selective-attention task served to bias selective attention towards the temporal regularities in the attended-colour stream, which allowed us to investigate whether selective attention modulates visual statistical learning. Visual statistical learning of temporal regularities in this task can be considered difficult. This is because stimulus repetitions were added to the attended-colour stream (as opposed to the stimulus repetition being the first stimulus of the next triplet in the attended-colour stream) and the presentation of stimuli in the attended-colour stream was interleaved with the presentation of stimuli in the unattended-colour stream. In the selective-attention task, participants’ number of stimulus-repetition detections, response time to stimulus-repetition detection, and number of false alarms were recorded. Stimulus-repetition detections were only considered to be a valid response if they were made within 3,000 ms from the onset of the stimulus repetition. The familiarisation phase took approximately 12 min to complete.

#### Triplet-discrimination task in the test phase

Immediately after the familiarisation phase, participants completed the test phase, which involved a 2IFC triplet-discrimination task. Participants were informed that in this phase, the stimuli presented would be coloured black, unlike in the familiarisation phase where the stimuli presented were in the colours green or red. Participants were also informed that on each trial, two triplets would be presented sequentially. Triplet Sequence 1 consisted of each of the three stimuli in this triplet, presented individually for 800 ms (same stimulus presentation duration as each stimulus in the familiarisation phase). This triplet sequence presentation was followed by a 1,000 ms interval, and then Triplet Sequence 2 was presented, which also consisted of each of the three stimuli in this triplet presented individually for 800 ms. Participants were asked to indicate which one of the two triplets was more familiar (“Which is the more familiar sequence? Sequence 1 or Sequence 2”), based on the information from the familiarisation phase. This triplet-discrimination task is a 2IFC task because, on each trial, the participants make their choice between two triplets that were presented sequentially, with a 1,000 ms interval between them. On each trial, one of the two triplets was a familiar triplet that had been presented in either the attended-colour stream (“attended triplet”) or the unattended-colour stream (“unattended triplet”). The other triplet was an impossible triplet that had never been presented in the familiarisation phase but was made up of stimuli from familiar triplets either in the attended-colour or unattended-colour stream. Each impossible triplet was made up of stimuli from either three different attended triplets or three different unattended triplets. Each stimulus in the impossible triplet was presented at the same triplet position as when it appeared in a familiar triplet. For example, stimulus *D* was presented at the first triplet position in both the familiar and impossible triplets (familiar triplet, *D–E–F* and impossible triplet, *D–B–I*).

In the triplet-discrimination task, there were eight familiar triplets (four attended triplets and four unattended triplets). There were also eight impossible triplets—four impossible triplets made up of stimuli from the attended-colour stream (each triplet was made up of stimuli from three different attended triplets) and four impossible triplets made up of stimuli from the unattended-colour stream (each triplet was made up of stimuli from three different unattended triplets). Each of the four familiar attended triplets was paired with each of the four impossible triplets (that were made up of stimuli from different attended triplets), and this pairing was presented twice. There were 32 attended-triplet-discrimination trials (i.e., four familiar triplets × four impossible triplets × two repetitions). Similarly, each of the four familiar unattended triplets was paired with each of the four impossible triplets (that were made up of stimuli from different unattended triplets), and this pairing was presented twice. There were 32 unattended-triplet-discrimination trials (i.e., four familiar triplets × four impossible triplets × two repetitions). In total, there were 64 trials in the triplet-discrimination task of the test phase. The order of trial presentation was randomised for each participant. Participants’ responses for attended-triplet and unattended-triplet discrimination in the test phase were recorded separately. Performance on this task was used as the measure for visual statistical learning.

**Figure 2.**
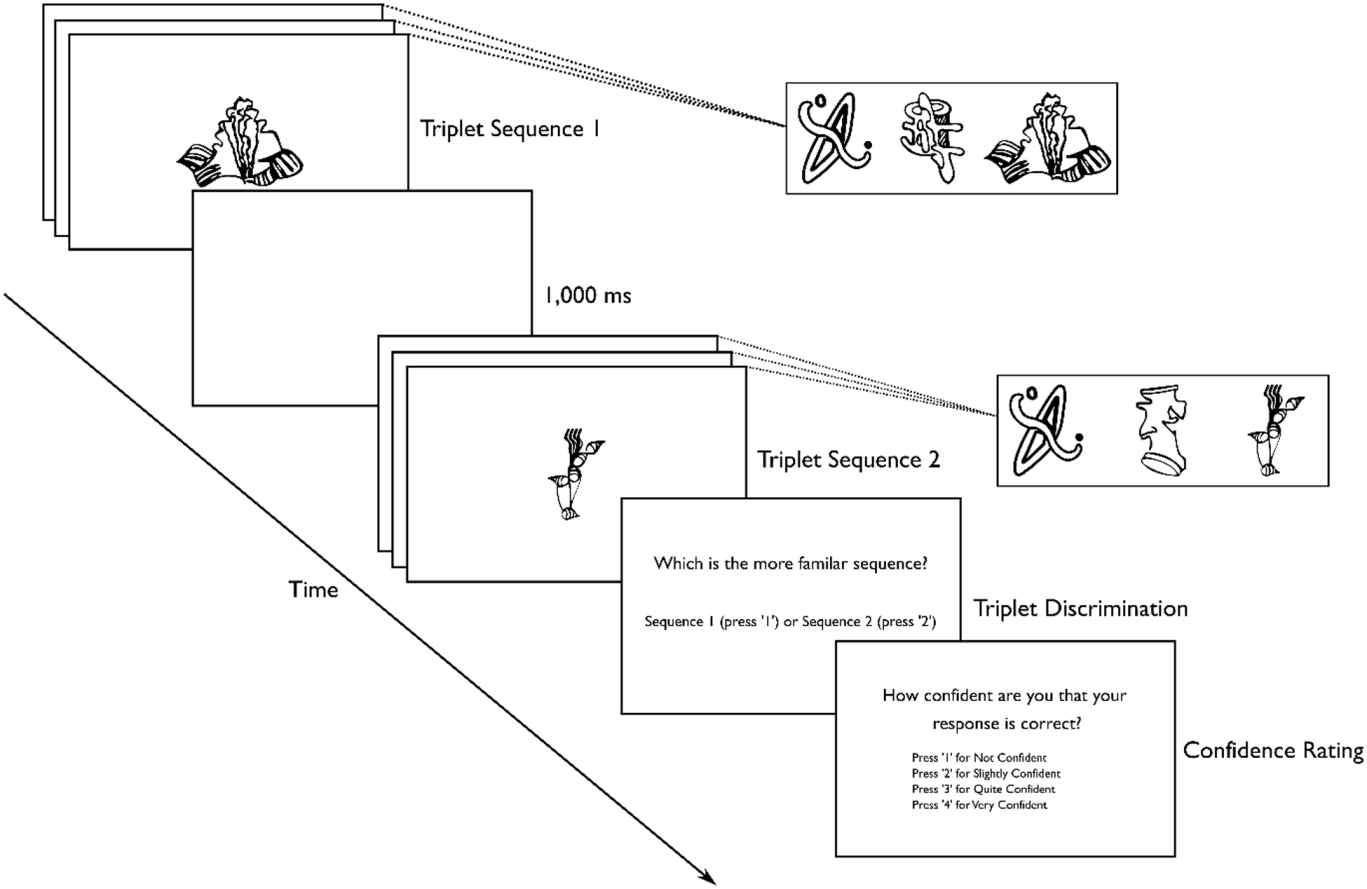
Example of a triplet-discrimination trial in the test phase of the visual statistical learning task. Participants were asked to complete a triplet-discrimination task in the test phase. They were informed that on each trial, two triplets would be presented sequentially. Triplet Sequence 1 consisted of each of the three stimuli in this triplet presented individually for 800 ms (same stimulus presentation duration as each stimulus in the familiarisation phase). This triplet sequence presentation was followed by a 1,000 ms interval, and then Triplet Sequence 2 was presented, which also consisted of each of the three stimuli in this triplet presented individually for 800 ms. Participants were asked to indicate which one of the two triplets was more familiar (“Which is the more familiar sequence? Sequence 1 or Sequence 2”), based on the information from the familiarisation phase. One of the two triplets was a familiar triplet that had been presented in either the attended-colour stream (“attended triplet”) or the unattended-colour stream (“unattended triplet”). The other triplet was an impossible triplet that had never been presented in the familiarisation phase but was made up of stimuli from familiar triplets either in the attended-colour or unattended-colour stream. After each triplet discrimination, participants were asked to rate their confidence that the response for the triplet discrimination was correct (“How confident are you that your response is correct?”) on a 4-point rating scale. In the actual task, the 4-point confidence-rating scale was presented as a single line.

After each triplet discrimination, participants were asked to rate their confidence that the response for the triplet discrimination was correct (“How confident are you that your response is correct?”). Their confidence was rated using a 4-point scale: 1 = *not confident*; 2 = *slightly confident*; 3 = *quite confident*; and 4 = *very confident*. This confidence-rating scale was administered to assess participants’ awareness of the regularities in the visual statistical learning task. Figure 2 illustrates an example of a trial in the test phase. There were no time constraints for responding to the triplet-discrimination and confidence-rating tasks. The test phase took approximately 10 min to complete.

### Post-experiment questionnaire

Following their completion of the visual statistical learning task, participants were asked to complete a brief, online Post-Experiment Questionnaire. The online questionnaire was programmed using Qualtrics survey software. The purpose of this questionnaire was three-fold. We assessed whether participants (1) noticed any regularities in the familiarisation phase; (2) named or labelled the stimuli during the familiarisation phase; and (3) adopted strategies during the familiarisation phase to help them complete the task.

#### Noticing regularities in the familiarisation phase of the visual statistical learning task

Participants’ awareness of regularities in the familiarisation phase of the visual statistical learning task was assessed using the 4-point confidence-rating scale that was administered alongside each triplet discrimination in the test phase. We proposed that the 4-point confidence-rating scale is a more appropriate assessment of awareness. In addition, in line with many existing studies on visual statistical learning, which used a self-report measure after the completion of the visual statistical learning task to assess awareness of regularities (e.g., Otsuka et al., 2013; Turk-Browne et al., 2005), we administered a self-report measure after the completion of the visual statistical learning task in our Post-Experiment Questionnaire.

The purpose of the first part of the Post-Experiment Questionnaire was to assess whether participants noticed regularities in the familiarisation phase of the task, using the self-report measure. More specifically, participants were asked:

Were there any patterns in the sequence of objects in the first part of the task where the objects were shown to you in green and red? Please think carefully before you respond yes or no, as we are interested in knowing whether any patterns in the sequence of objects were obvious to you before you did the second part of the task where the objects were shown to you in black.

If participants indicated that they did notice regularities in the task, we asked them to describe the regularities they noticed in the familiarisation phase (“Please describe the pattern(s) you noticed in the sequence of objects in the first part of the task where the objects were shown to you in green and red”).

#### Naming or labelling stimuli during the familiarisation phase of the visual statistical learning task

The purpose of the second part of the Post-Experiment Questionnaire was to assess whether participants named or labelled the stimuli during the familiarisation phase of the visual statistical learning task. Participants were not instructed to name or label any of the stimuli during the familiarisation phase, but we found in previous studies conducted in our laboratory (Cox & Aimola Davies, 2022; Cox et al., 2022) that participants named or labelled the stimuli, even if the stimuli were considered not to be nameable (e.g., abstract shapes). In this part of the questionnaire, participants had the opportunity to make a number of different responses. For each stimulus, participants were asked if they named or labelled the stimulus to help them complete the familiarisation phase (“Did you name or label the object to help you complete the first part of the task?”). If participants indicated that they did name or label the given stimulus, they were asked to provide the name or label they applied, or indicate if they had forgotten the name or label they applied. If participants indicated that they did not name or label the given stimulus, they were asked to indicate if they had recognised the stimulus but did not apply a name or label to it, or if they had not recognised the stimulus.

#### Adopting strategies during the familiarisation phase of the visual statistical learning task

The purpose of the third and final part of the Post-Experiment Questionnaire was to assess whether participants adopted strategies during the familiarisation phase of the visual statistical learning task, to help them complete the task (“Did you use strategies [other than naming or labelling of the objects] to help you complete the first part of the task?”). If participants indicated that they used strategies (other than naming or labelling of the stimuli), we asked them to describe these strategies (“Please describe the strategies you have used [other than naming or labelling of the objects] to help you complete the first part of the task”).

### Statistical analysis plan

Generalised linear mixed-effects models were used to conduct the main analyses. All analyses were carried out in R4.1.2, with the *lme4* package for generalised linear mixed-effects models (Bates et al., 2015), *car* package for Type-III analysis of variance (ANOVA) tables with Wald chi-square tests (Fox & Weisberg, 2019), and *emmeans* package for post hoc comparisons with Tukey’s corrections for significance levels (Lenth, 2022; Searle et al., 1980). Estimated marginal means for effects were reported in our results. Unless explicitly stated, fixed effects were entered into the regression models using deviation coding, where the intercept represents the grand mean, and the coefficients represent the main effects. Deviation coding is used to enable ANOVA-style inferences.

## Results

### Selective-attention task in the familiarisation phase

We analysed performance on the selective-attention task in the familiarisation phase to ensure that participants completed the task as instructed, irrespective of the animacy condition (unattend-animate, unattend-inanimate) they completed.

Participants’ trial-level stimulus-repetition detection accuracy (0 = *miss*, 1 = *hit*) was analysed using a mixed-effects logistic regression. The factor, animacy condition (unattend-animate, unattend-inanimate) was entered into the regression model as a fixed effect, and participant (i.e., individual participant) was entered into the model as a random intercept.^1^ The model indicated that stimulus-repetition detection accuracy did not differ between the animacy conditions. The main effect of animacy condition was not significant, χ^2^(1) = 0.079, *p* = .779. The mean stimulus-repetition detection accuracy (proportion correct) for participants in the unattend-animate condition was 0.97, *SE* = 0.01, 95% CI = [0.93, 0.98], and for participants in the unattend-inanimate condition, it was 0.96, *SE* = 0.01, 95% CI = [0.92, 0.98].

Participants’ trial-level stimulus-repetition response time (ms) was analysed using a mixed-effects gamma regression. The factor, animacy condition (unattended-animate, unattended-inanimate) was entered into the regression model as a fixed effect, and participant (i.e., individual participant) and stimulus-repetition item (i.e., the stimulus repetition presented in the familiarisation phase) were entered into the model as random intercepts. The model indicated that stimulus-repetition detection response times did not differ between the animacy conditions. The main effect of animacy condition was not significant, χ^2^(1) = 2.524, *p* = .112. The mean stimulus-repetition response times for participants in the unattend-animate condition was 611.68 ms, *SE* = 12.62, 95% CI = [586.92, 636.44], and for participants in the unattend-inanimate condition, it was 589.79 ms, *SE* = 12.23, 95% CI = [565.80, 613.79].

### Triplet-discrimination task in the test phase

#### Triplet-discrimination performance

We analysed performance on the triplet-discrimination task in the test phase to determine the effects of animacy on visual statistical learning of attended and unattended information.

Participants’ trial-level triplet discrimination (0 = *incorrect*, 1 = *correct*) was analysed using a mixed-effects logistic regression. The factors, animacy condition (unattend-animate, unattend-inanimate), and visual stream (attended, unattended) were entered into the regression model as fixed effects. Visual stream (attended, unattended) was also entered into the model as a random slope, and participant (i.e., individual participant) was entered as a random intercept.^2^

The model revealed that the main effect of animacy was not significant, χ^2^(1) = 0.259, *p* = .611 and the animacy by visual stream interaction was not significant, χ^2^(1) = 0.423, *p* = .516. The main effect of visual stream was significant, χ^2^(1) = 20.317, *p* < .001. Irrespective of animacy condition, there was better performance for attended-triplet discrimination, *M* = 0.66, *SE* = 0.03, 95% CI = [0.60, 0.71], compared with performance for unattended-triplet discrimination, *M* = 0.53, *SE* = 0.01, 95% CI = [0.50, 0.55]. We ran further analyses using null models (i.e., no fixed factors entered into the model) to determine whether performance for attended-triplet and unattended-triplet discrimination was above chance (0.50), which would suggest that there was visual statistical learning of attended and unattended information. Because logit of 0.5 is 0, a positive intercept in a null model would indicate that triplet discrimination was above chance, meaning that visual statistical learning occurred. The intercept for the null model for attended-triplet discrimination was significant and positive, χ^2^(1) = 27.831, *p* < .001. The intercept for the null model for unattended-triplet discrimination was also significant and positive, χ^2^(1) = 4.263, *p* = .040. Results from these null models indicated that performance for attended-triplet and unattended-triplet discrimination was above chance, meaning that there was visual statistical learning of attended and unattended information.

**Figure 3.**
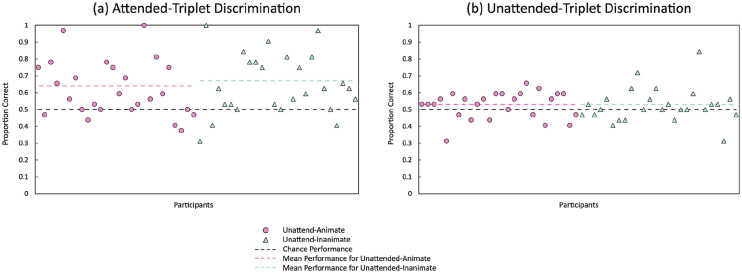
Performance on the triplet-discrimination task in the test phase for attended-triplet and unattended-triplet discrimination. The scatterplots illustrate participants’ performance (proportion correct) for the discrimination of (a) familiar attended triplets from impossible triplets (attended-triplet discrimination) and (b) familiar unattended triplets from impossible triplets (unattended-triplet discrimination). Each point represents one participant. The pink circles represent participants who completed the visual statistical learning task with animate line-drawings in the unattended visual stream (unattend-animate condition). The green triangles represent participants who completed the visual statistical learning task with inanimate line-drawings in the unattended visual stream (unattend-inanimate condition). The dashed black line represents chance performance (0.50) in the two-interval forced-choice triplet-discrimination task. The dashed pink line represents mean triplet-discrimination performance (estimated marginal means) for the unattend-animate condition. The dashed green line represents mean triplet-discrimination performance (estimated marginal means) for the unattend-inanimate condition. The analyses indicated that, overall for attended-triplet discrimination, participants performed above chance (i.e., demonstrated visual statistical learning of attended information). Similarly, overall for unattended-triplet discrimination, participants performed above chance (i.e., demonstrated visual statistical learning of unattended information). Across both attended- and unattended-triplet discrimination, performance did not differ between the two animacy conditions tested. Please refer to the online version of the article for the colour version of this figure.

Although the animacy by visual stream interaction was not significant, one of our hypotheses was that visual statistical learning of *attended* information would differ between the two animacy conditions (unattend-animate, unattend inanimate), such that, there would be poorer visual statistical learning for attended information by participants in the unattend-animate condition compared with participants in the unattend-inanimate condition. We conducted a post hoc comparison for attended-triplet discrimination between participants in the unattend-animate condition and those in the unattend-inanimate condition to ascertain whether the hypothesis was supported. The post hoc comparison indicated that triplet-discrimination performance did not differ between the two conditions, *p* = .558, unattend-animate: *M* = 0.64, *SE* = 0.04, 95% CI = [0.56, 0.71]; unattend-inanimate: *M* = 0.67, *SE* = 0.04, 95% CI = [0.59, 0.74]. Our other hypothesis was that visual statistical learning of *unattended* information would differ between the two animacy conditions (unattend-animate, unattend-inanimate), such that, there would be greater visual statistical learning for unattended information by participants in the unattend-animate condition compared with participants in the unattend-inanimate condition. We conducted a post hoc comparison for unattended-triplet discrimination between participants in the unattend-animate condition and those in the unattend-inanimate condition to ascertain whether the hypothesis was supported. The post hoc comparison indicated that triplet-discrimination performance did not differ between the two conditions, *p* = .962, unattend-animate: *M* = 0.53, *SE* = 0.02, 95% CI = [0.49, 0.56]; unattend-inanimate: *M* = 0.53, *SE* = 0.02, 95% CI = [0.49, 0.56]. The scatterplots for individual attended-triplet-discrimination performance and unattended-triplet-discrimination performance are presented in Figure 3.

#### Confidence ratings for triplet discrimination

We analysed confidence ratings for triplet discrimination to examine participants’ awareness of the regularities in the familiarisation phase of the visual statistical learning task. Participants’ knowledge of the regularities in the visual statistical learning task is considered to be under the subjective threshold of consciousness if the following two criteria are met: the guessing criterion and the zero-correlation criterion (Cheesman & Merikle, 2001; Dienes, 2004). The guessing criterion is met if participants demonstrate above-chance performance on the triplet-discrimination task despite reporting being not confident (or guessing) of their responses for triplet discrimination. The zero-correlation criterion is met if participants’ confidence of their responses for triplet discrimination is not correlated with their performance on the triplet-discrimination task. We analysed confidence ratings for attended- and unattended-triplet discrimination separately. But note that, in previous studies conducted in our laboratory (Cox & Aimola Davies, 2022; Cox et al., 2022), we have not found any relationship between confidence ratings and unattended-triplet discrimination. Participants’ trial-level triplet discrimination (0 = *incorrect*, 1 = *correct*) was analysed using a mixed-effects logistic regression. The factor, confidence rating (1 = *not confident*; 2 = *slightly confident*; 3 = *quite confident*; 4 = *very confident*) was entered into the model as a fixed effect, and participant (i.e., individual participant) was entered as a random intercept. Dummy coding was used for confidence rating (comparing each level to the reference level), with the lowest rating (1 = *not confident*) used as the reference level.

The model for attended-triplet discrimination revealed that confidence rating was a significant predictor for attended-triplet-discrimination performance, χ^2^(3) = 60.947, *p* < .001. To assess whether the guessing criterion was met, we referred to the regression intercept. This is because the lowest rating (1 = *not confident*) was used as the reference level, and since logit 0.5 equals 0, a significant positive intercept would indicate that attended-triplet-discrimination performance was above chance when the lowest rating was used. The model indicated that the intercept was not significantly positive, *b* = 0.019, *SE* = 0.15, *z* = 0.130, *p* = .896, suggesting that triplet-discrimination performance was not above chance when participants reported being not confident. This result means that the guessing criterion was not met. To assess whether the zero-correlation criterion was met, we ran pairwise comparisons on the model coefficients to determine whether attended-triplet-discrimination performance increased as confidence rating increased. Pairwise comparisons indicated that overall attended-triplet-discrimination performance increased as confidence rating increased (see Table 1). This result means that the zero-correlation criterion was not met. The model for unattended-triplet discrimination indicated that confidence rating for unattended-triplet discrimination was not a significant predictor for unattended-triplet-discrimination performance, χ^2^(3) = 3.090, *p* = .378.

**Table 1.** Pairwise comparisons of regression coefficients for attended-triplet discrimination for the confidence ratings.

Confidence ratings	Estimate difference	*SE*	*p*
1 = *not confident* versus 2 = *slightly confident*	–0.351	0.150	.088
1 = *not confident* versus 3 = *quite confident*	–0.845	0.170	<.001
1 = *not confident* versus 4 = *very confident*	–1.727	0.242	<.001
2 = *slightly confident* versus 3 = *quite confident*	–0.494	0.141	.003
2 = *slightly confident* versus 4 = *very confident*	–1.376	0.224	<.001
3 = *quite confident* versus 4 = *very confident*	–0.882	0.225	<.001

SE: standard error.

Estimates are given on the log odds ratio scale.

### Post-experiment questionnaire

#### Noticing regularities in the familiarisation phase of the visual statistical learning task

In the first part of the Post-Experiment Questionnaire, we asked participants to self-report whether they noticed any patterns or regularities in the familiarisation phase of the task. If they indicated that they noticed patterns or regularities in the familiarisation phase, we asked them to describe the patterns and regularities they had noticed.

Thirty-four of 52 participants (65.38%) reported that they noticed patterns and regularities. Of these 34 participants, 16 participants were from the unattend-animate condition and 18 participants were from the unattend-inanimate condition. The qualitative descriptions provided by the 34 participants indicated that they were aware of the regularities in the line-drawings of non-objects (i.e., information they were asked to attend) in the familiarisation phase of the task despite not being informed of these regularities. Some of these participants indicated that they noticed the patterns or regularities, but they did not provide specific descriptions of these patterns or regularities. For example, “I only noticed the green. And though there are some repetitions, the shapes were shown in order” (participant from the unattend-animate condition who was asked to attend to green line-drawings of non-objects), or “When a sequence of green shapes [was] being shown, they appeared to follow a distinct pattern or one that was similar for each sequence” (participant from the unattend-inanimate condition who was asked to attend to green line-drawings of non-objects), or “I can recall that few patterns [for] red colour shape sequences repeated in the first part, usually three in a row. I cannot recall any patterns for green colour shape” (participant from the unattend-inanimate condition who was asked to attend to red line-drawings of non-objects). Whereas, other participants did provide specific descriptions of the patterns or regularities they noticed. For example, “the shape that looked like ‘seaweed’ was often followed by a shape I called ‘coral’” (participant from the unattend-animate condition who was asked to attend to red line-drawings of non-objects), or “The jumper, shirt, and undershirt squiggles (red shapes, as I named them), constantly appeared close to each other. The repeated shape was often the drum, which had two circles (top and bottom)” (participant from the unattend-inanimate condition who was asked to attend to red line-drawings of non-objects).

#### Naming or labelling stimuli during the familiarisation phase of the visual statistical learning task

In the second part of the Post-Experiment Questionnaire, we asked participants to self-report whether they named or labelled the stimuli during the familiarisation phase of the visual statistical learning task. Overall, it appears that naming or labelling of stimuli during the familiarisation phase of the visual statistical learning task was a common occurrence among participants. Across all participants, the mean proportion of stimuli named or labelled during the familiarisation phase was 0.64, *SD* = 0.48, meaning that on average, 15 of 24 stimuli were named or labelled during the familiarisation phase.

#### Adopting strategies during the familiarisation phase of the visual statistical learning task

In the third and final part of the Post-Experiment Questionnaire, we asked participants to self-report whether they adopted strategies (other than naming or labelling of the line-drawings) during the familiarisation phase of the visual statistical learning task, strategies they thought would help them complete the task. If they indicated that they adopted strategies to help them complete the task, we asked them to describe these strategies.

Fourteen of 52 participants (26.92%) reported that they adopted strategies (other than naming or labelling of the line-drawings) during the familiarisation phase of the visual statistical learning task. Of these 14 participants, 8 were from the unattend-animate condition and 6 were from the unattend-inanimate condition. The qualitative descriptions of these strategies included: “ignoring red objects completely” (participant from the unattend-animate condition who was asked to attend to green line-drawings of non-objects) and “remembering the special characteristic of the picture” (participant from the unattend-inanimate condition who was asked to attend to green line-drawings of non-objects). The mean proportion of stimuli named or labelled by participants who were in the unattend-animate and unattend-inanimate conditions, and the mean proportion of attended and unattended stimuli named or labelled during the familiarisation phase, are displayed in Table 2.

**Table 2.** Mean proportion of stimuli named or labelled in the unattend-animate and unattend-inanimate conditions.

Animacy condition	Visual stream	*M*	*SD*
Unattend-animate	Attend	0.974	0.158
	Unattend	0.401	0.491
Unattend-inanimate	Attend	0.917	0.277
	Unattend	0.276	0.448

SD: standard deviation.

## Discussion

In the present study, we examined the effects of animacy on visual statistical learning of temporal regularities. We manipulated the animacy status of the unattended information in our visual statistical learning task, to determine whether this animacy manipulation would affect visual statistical learning of attended and unattended information. We tested two animacy conditions—animates (i.e., animals) and inanimates (i.e., tools and kitchen utensils). We also examined whether visual statistical learning occurs with or without awareness of the temporal regularities. There were four key findings. The first finding is that selective attention modulates visual statistical learning. Consistent with previous studies in our laboratory group (e.g., Cox & Aimola Davies, 2022; Cox et al., 2022) and those of other researchers (e.g., Turk-Browne et al., 2005), there was greater visual statistical learning for attended information than for unattended information. The second finding is that animacy does not affect visual statistical learning. We found visual statistical learning in the two animacy conditions tested (unattend-animate, unattend-inanimate), and visual statistical learning of attended and unattended information did not differ between the two animacy conditions. The third finding is that visual statistical learning occurs with awareness of regularities. The 4-point confidence ratings for triplet discrimination indicated that participants were aware of regularities in the attended visual stream of the visual statistical learning task. This finding was also supported by their self-report descriptions that revealed awareness of the regularities. The fourth finding is that strategies (including naming and labelling of the line-drawings) were used during the familiarisation phase of the visual statistical learning task, and that this use of strategies was common among our participants. In the following subsections, we further discuss these four key findings.

### Selective attention modulates visual statistical learning

We manipulated selective attention by asking participants to attend to stimuli in one of two visual streams and to detect stimulus repetitions in that visual stream (i.e., a stimulus that was identical to the last stimulus they observed in that same stream). Consistent with previous studies in our laboratory group (e.g., Cox & Aimola Davies, 2022; Cox et al., 2022) and those of other researchers (e.g., Turk-Browne et al., 2005), we found greater visual statistical learning for attended information than for unattended information. Participants demonstrated better performance for attended-triplet discrimination than for unattended-triplet discrimination. This finding—that selective attention modulates visual statistical learning—is not surprising given the prominent role that selective attention plays in visual cognition and perception. It is important to note that the relationship between selective attention and visual statistical learning is not uni-directional, but rather bi-directional. Recent studies have shown that visual statistical learning of regularities (e.g., regularities in distractor location) can modulate attentional selection (e.g., Duncan & Theeuwes, 2020; Huang et al., 2021; Li & Theeuwes, 2020). Li and Theeuwes (2020) proposed that learning statistical regularities in a visual search task results in more efficient target selection, suggesting that through visual statistical learning, “. . . the weights, within the spatial priority map, are dynamically adapted such that following the selection of one location (the predicting location), on the next trial, the weight of another location (the predicted location) is up-regulated” (p. 9).

In our study, we found visual statistical learning of both attended and unattended information. Participants demonstrated above-chance performance for attended-triplet discrimination and unattended-triplet discrimination. We emphasise here that visual statistical learning is modulated by selective attention, rather than that visual statistical learning is *gated* by selective attention (meaning that, without selective attention, visual statistical learning would not occur). Findings from the present study, and from our previous study (Cox et al., 2022), suggest that visual statistical learning can occur for unattended information (but the size of this effect may be small). There are several possible explanations as to why visual statistical learning of unattended information occurs.

The first possible explanation relates to stimulus- and task-related factors. In our previous study (Cox et al., 2022), in which we observed visual statistical learning of unattended information, we used highly-familiar line-drawings from the same semantic category in both the attended and unattended visual streams in our visual statistical learning task. We suggested that this task design may have resulted in poorer inhibition of unattended information, resulting in visual statistical learning of unattended information. Exploratory analyses in that study indicated that only participants who did not demonstrate visual statistical learning of attended information demonstrated visual statistical learning of unattended information. However, this explanation does not appear to be a valid explanation for the results in the present study because we observed visual statistical learning of unattended information even when the semantic category of the line-drawings used in the attended visual stream was different from the semantic category of the line-drawings used in the unattended visual stream. It remains possible that other stimulus- and task-related factors resulted in visual statistical learning of unattended information.

The second possible explanation relates to the administration of a brief practice exercise for the selective-attention task. Visual statistical learning of unattended information was observed in studies that have administered a brief practice exercise for the selective-attention task (Cox et al., 2022; Musz et al., 2015; Experiment 4) but not in studies that have not administered a brief practice exercise for the selective-attention task (Turk-Browne et al., 2005). The practice exercise may have been so effective and successful at familiarising participants with the selective-attention task that participants then had additional attentional resources that “spilled over” to the unattended information (Lavie, 2010; Lavie et al., 2004 and see also Musz et al., 2015), thus facilitating visual statistical learning of unattended information. We believe that the administration of the brief practice exercise in the current study may have reduced the perceptual load for the observer during the selective-attention task. According to perceptual load theory (Lavie et al., 2014), observers have a limited capacity for perceptual processing, such that, the maximum amount of information that falls within this capacity will be processed automatically and involuntarily. Selective attention allows observers to set a top-down priority, such that, task-relevant information is given higher priority for attentional resources compared with task-irrelevant information. When the task assigned to the observer is difficult (i.e., the perceptual load is high), there should be little or no capacity remaining once task-relevant information has been processed, thus leaving no capacity for task-irrelevant information. When the task assigned to the observer is easy (i.e., the perceptual load is low), there will be residual capacity remaining once task-relevant information has been processed. And, since processing is automatic and involuntary, this residual capacity will be used for processing task-irrelevant information even if it was supposed to have been ignored. In the context of visual statistical learning research, we believe that when the perceptual load of the visual statistical learning task is low, observers have the residual capacity required to process task-irrelevant information (e.g., the unattended information in the visual statistical learning task).

The third possible explanation relates to the sample size of the study. There was no evidence for visual statistical learning of unattended information when the study involved smaller sample sizes (e.g., *N* = 8 in the Turk-Browne et al., 2005 study), but there was evidence for visual statistical learning of unattended information when the study involved larger sample sizes (e.g., *N* = 52 in the present study and *N* = 284 in the Cox et al., 2022 study). It is well accepted that studies with smaller sample sizes have less statistical power to detect a true effect (VanVoorhis & Morgan, 2007). Thus, having a small sample size may have obscured the ability to detect visual statistical learning of unattended information in previous research. However, we note here that although these two studies with larger sample sizes (the present study and the Cox et al., 2022 study) have reported evidence for visual statistical learning of unattended information, the size of the effect observed was small.

The fourth explanation relates to the information that the participants attended to during the familiarisation phase. In the present study, we manipulated selective attention in the familiarisation phase of the visual statistical learning task by instructing participants to attend either to the green- or red-coloured stream (i.e., the attended visual stream) and to respond to stimulus repetitions in this attended visual stream. Participants demonstrated high accuracy in detecting the stimulus repetitions in the attended visual stream, suggesting that participants were attending to the visual stream that they were instructed to attend. This approach is also commonly adopted in existing literature on visual statistical learning. However, it is possible that the participants may have occasionally attended to stimuli in the visual stream they were not asked to attend to (i.e., the unattended visual stream) during the familiarisation phase. Future research should consider improving this study design so that it is possible to assess whether the participants have been attending only to the visual stream that they were instructed to attend. For example, one could incorporate eye-tracking within a selective-attention task, and attempt to separate the location in which the two visual streams are presented so as to make it difficult to fixate on both visual streams simultaneously. If participants’ eye-tracking shows that they are fixated on the visual stream that contains the attended information, one could suggest that participants are attending to the visual stream as instructed.

More broadly in statistical learning research, there is also evidence for *auditory* statistical learning in the absence of selective attention (e.g., Batterink & Paller, 2019; Saffran et al., 1997). A good demonstration of this finding would be the study by Saffran et al. (1997). In their study, both children and adults demonstrated auditory statistical learning of an artificial language despite their attention being engaged in the primary task of creating computer illustrations. More specifically, participants were not told that they would be listening to an artificial language, or even that they should listen. Yet, despite their attention being engaged in creating computer illustrations, they demonstrated auditory statistical learning of the artificial language. These findings for auditory statistical learning—that there is evidence for auditory statistical learning in the absence of selective attention—are consistent with our finding that visual statistical learning can also occur in the absence of selective attention.

### Animacy does not affect visual statistical learning

We manipulated the animacy status of unattended information in our visual statistical learning task, to determine whether this animacy manipulation would affect visual statistical learning of attended and unattended information. The two animacy conditions tested were animates (unattend-animate; i.e., animals) and inanimates (unattend-inanimate; i.e., tools and kitchen utensils). In both of these animacy conditions, we found that overall there was visual statistical learning. However, animacy did not affect visual statistical learning of either the attended or unattended information. There were no differences between the two animacy conditions in attended-triplet and unattended-triplet discrimination. We point out here that in our previous study (Cox et al., 2022), where we manipulated the animacy status of attended *and* unattended information in our visual statistical learning task, we also did not observe any effects of animacy on visual statistical learning.

The lack of any effects of animacy on visual statistical learning in the present study and in our previous study (Cox et al., 2022) may be attributed to task-related factors, such as the study design and the stimulus presentation duration, or to other stimulus-related factors. The study design we adopted in the present study (where we manipulated only the animacy status of unattended information) and the study design we adopted in our previous study (where we manipulated the animacy status of attended and unattended information) were both between-subjects designs, which may not be adequately sensitive to detect any effects of animacy on visual statistical learning. Studies investigating animacy effects in perception and cognition (with experimental paradigms other than visual statistical learning) have typically used a within-subjects design (e.g., Altman et al., 2016; Bonin et al., 2014; Bugaiska et al., 2019), and these studies have reported strong effects of animacy, such that, stimuli that belonged to an animate category received preferential processing over stimuli that belonged to an inanimate category. Future research investigating the effects of animacy on visual statistical learning should consider the use of a within-subjects design. For example, one could consider presenting triplets made up of animate stimuli and triplets made up of inanimate stimuli within one visual stream in the visual statistical learning task to determine whether triplets made up of animate stimuli are learned better than triplets made up of inanimate stimuli. The long stimulus presentation duration (each stimulus is presented for 800 ms) and frequency (each stimulus is presented 24 times) in our visual statistical learning task may also have rendered the task insensitive to detect any effects of animacy.

The lack of any effects of animacy may also be attributed to stimulus-related factors, such as the use of line-drawing stimuli in the present study. The line-drawing stimuli used in the present study were from the pictorial database by Snodgrass and Vanderwart (1980), which has been used extensively in experimental psychology research to evoke representations of a concept (e.g., animate or inanimate entities). However, these line-drawings do not contain features that are important in object recognition processes (e.g., colour, texture). Future research investigating the effects of animacy on visual statistical learning should consider using object stimuli (e.g., Bank of Standardised Stimuli by Brodeur et al., 2010) that potentially may evoke better representations of animate and inanimate entities (but see studies by Hagen & Laeng, 2016, 2017).

It remains an open, empirical question as to whether manipulating the content of unattended information in the visual statistical learning task would have any effect on visual statistical learning more broadly. One could test this research question by manipulating the salience of information in the unattended visual stream.^3^ For example, one could manipulate the salience of information in the unattended visual stream by presenting participants with faces, instead of the animate or inanimate stimuli we used in the present study. Salience could be explored further by presenting one group of participants with happy faces in the unattended visual stream of the familiarisation phase of the visual statistical learning task, and presenting another group of participants with angry faces. To ensure that the perceptual load of the proposed study is consistent with that of the present study, one would present participants with line-drawings of non-objects in the attended visual stream of the familiarisation phase. Given that happy or angry faces are more emotionally-salient when compared with neutral faces (e.g., Thomas et al., 2014), there may be greater visual statistical learning of unattended information when emotionally-salient faces are presented in the unattended visual stream than when neutral faces are presented in the unattended visual stream. If this effect is observed, then one could suggest that emotional salience affects visual statistical learning but that animacy does not. However, if this effect is not observed, then one could suggest that the content of unattended information may not affect visual statistical learning, irrespective of the type of content being presented in the unattended visual stream.

Beyond these methodological concerns, we must not ignore the possibility that on a conceptual level, animacy may not affect visual statistical learning. Evolutionary psychologists proposed that our visual attentional system has evolved to monitor and select information (i.e., animates) that signal opportunities and dangers. The ability to detect animates (as compared with inanimates) rapidly is advantageous for human survival and was therefore thought to be a consequence of our ancestral hunter-gatherer background. Evolutionary psychologists have also proposed that other aspects of cognition are specialised and functionally organised to solve adaptive problems (see review by Cosmides & Tooby, 2013). There is evidence supporting the notion that statistical learning is a critical function in cognition as it underlies a range of behaviours that are relevant throughout the lifespan (see review by Sherman et al., 2020), such as learning a language (e.g., Saffran et al., 1996), learning and planning motor behaviours (e.g., Monroy et al., 2019) and more broadly, generating predictions about upcoming experiences (e.g., Creel et al., 2004; Kirkham et al., 2002; Moldwin et al., 2017; Morgan et al., 2019). Given the prevalence and importance of statistical learning in cognition, one could assume that being able to extract regularities in how animates are likely to behave or where animates are likely to appear would be highly advantageous for human survival. However, the prevalence and importance of statistical learning in cognition could also mean that statistical learning mechanisms are not influenced by the stimulus category of information (including animacy categories) because statistical learning of information from any stimulus category is important. Indeed, in another study of ours (Cox & Aimola Davies, 2022), we did not find any effects of stimulus category on visual statistical learning when we compared visual statistical learning of abstract shapes with visual statistical learning of highly-familiar line-drawings.

### Visual statistical learning occurs with awareness of regularities

We measured participants’ awareness of regularities in the visual statistical learning task by implementing an assessment of awareness (4-point confidence-rating scale) after each triplet discrimination. Using the 4-point confidence-rating scale, we found that the guessing criterion was not met. This means that participants’ performance for attended-triplet discrimination was not above chance when they reported being not confident of their triplet-discrimination response. We also found that the zero-correlation criterion was not met. This means that participants’ performance for attended-triplet discrimination increased as their confidence in their triplet-discrimination responses increased. Participants’ knowledge of the regularities in the attended visual stream of the visual statistical learning task is thus considered not to be under the subjective threshold of consciousness (Cheesman & Merikle, 2001; Dienes, 2004). Our findings are generally consistent with previous studies that have implemented an assessment of awareness after each triplet discrimination in the visual statistical learning task (e.g., Bertels et al., 2012; Cox & Aimola Davies, 2022; Cox et al., 2022; and see also Wierzchoń et al., 2012), indicating that visual statistical learning of attended information occurred with awareness of statistical regularities.

Other studies in visual statistical learning have used self-report measures administered after participants completed the entire visual statistical learning task (i.e., after the test phase) to assess awareness of regularities (e.g., Otsuka et al., 2013). The general findings from these studies indicate that participants were not aware of statistical regularities in the task. In the present study, in addition to measuring participants’ awareness of regularities using the 4-point confidence-rating scale after each triplet-discrimination trial, we administered a Post-Experiment Questionnaire, in which we measured participants’ awareness of regularities by asking them to self-report whether they noticed regularities and to provide descriptions of these regularities. We found that more than half of our participants were aware of statistical regularities, with some participants reporting that they noticed “repetitions” or “sequences” in the familiarisation phase (in particular, repetitions or sequences in the attended visual stream), while other participants reported and described these repetitions or sequences. These self-report responses complement our findings from the 4-point confidence-rating scale, such that, they support the finding that visual statistical learning of attended information occurs with awareness of statistical regularities. The overall findings from the present study suggest that previous studies may have underestimated the contributions of awareness to visual statistical learning, and they highlight that careful consideration is required in how awareness of regularities is assessed in studies on visual statistical learning.

Although we found evidence that participants were aware of regularities in the attended visual stream of the visual statistical learning task, we were unable to assess whether participants were aware of regularities in the *unattended* visual stream. That is, we were unable to assess whether the guessing criterion or the zero-correlation criterion were met for regularities in the unattended visual stream (based on unattended-triplet discrimination). We suggest that this was because participants’ performance for unattended-triplet discrimination was clustered around chance level (floor effects). Participants’ responses to the self-report measure administered in the Post-Experiment Questionnaire also indicated that the majority of participants did not notice any patterns or regularities in the unattended visual stream, as they were not attending to stimuli in the unattended visual stream.

Another important consideration is that the present study measured visual statistical learning using an offline measure. Visual statistical learning was measured in the test phase, after completion of the familiarisation phase. Previous researchers (e.g., Turk-Browne et al., 2005) have argued that the use of an offline measure may not be the most appropriate to measure visual statistical learning of what is believed to occur without conscious awareness. This is because, if visual statistical learning occurs without conscious awareness, the use of an offline measure that asks participants to make an explicit decision on information they have learned may underestimate participants’ visual statistical learning abilities. Researchers have suggested that the use of an offline implicit measure (e.g., Kim et al., 2009; Turk-Browne et al., 2005 but see Rawal & Tseng, 2021) or the use of an online measure (e.g., Sáringer et al., 2022) may be more appropriate to measure visual statistical learning given the historical claim that visual statistical learning occurs without conscious awareness. Future studies could incorporate an assessment of awareness (e.g., a 4-point confidence-rating scale) in an online visual statistical learning task to gain further insights on whether visual statistical learning occurs with or without conscious awareness.

### Strategies used during the familiarisation phase of the visual statistical learning task

We assessed whether participants had adopted any strategies during the familiarisation phase of the visual statistical learning task. More specifically, we asked participants if they had named or labelled the line-drawings during the familiarisation phase, and if they had used strategies (other than naming or labelling) during the familiarisation phase. Although participants had not been asked to name or label the line-drawings, or to use strategies, during the familiarisation phase of the visual statistical learning task, we found that naming and labelling of the line-drawings was a common occurrence among participants. Naming or labelling the line-drawings may be a strategy adopted by participants to help them “stay on task” during the familiarisation phase. Approximately one third of our participants also reported that they used strategies, other than naming or labelling, to help them complete the familiarisation phase of the visual statistical learning task. Some of these strategies included ignoring or not paying attention to line-drawings of animates or inanimates in the unattended visual stream, or remembering certain features of line-drawings of non-objects in the attended visual stream.

We were unable to ascertain whether the use of strategies—naming or labelling of stimuli or other strategies—during the familiarisation phase affected visual statistical learning, as we did not systematically manipulate these strategies or measure the use of these strategies during the familiarisation phase. The data collected in this study do provide insights into whether participants are using strategies, but unfortunately the data collected does not provide further information either on when these strategies had been applied during the visual statistical learning task, or on how often these strategies had been applied. In addition, because the strategies applied by participants varied extensively, it remains unknown whether any of these different strategies lead to different visual statistical learning outcomes. We believe that in future we may be able to draw more meaningful conclusions on the effects of strategies on visual statistical learning by systematically manipulating and measuring the use of strategies.

We are not aware of any published studies that have systematically investigated how the use of strategies affects visual statistical learning (but see Himberger, 2021; Milne et al., 2018). Most relevant to this discussion point is the study by Bertels et al. (2015). Bertels et al. manipulated whether participants received information about the visual statistical learning task before they participated in the task. One group of participants, who received information about the visual statistical learning task, were informed about the presence of triplet sequences in the familiarisation phase, and that they would be asked to identify these triplet sequences after the familiarisation phase (in the test phase). The other group of participants did not receive any information about the presence of triplet sequences in the familiarisation phase or the nature of the test phase. Bertels et al. also manipulated the stimulus presentation duration (200 ms versus 800 ms). Participants who received information about the visual statistical learning task demonstrated better performance than participants who did not receive this information, but only under extended stimulus presentation duration (800 ms). Bertels et al. suggested that participants who received information about the visual statistical learning task were able to “set up explicit strategies in order to extract the statistical regularities” (p. 6) when the stimulus presentation duration was extended. They did not ask participants about the strategies they had adopted but they speculated that the strategies may have consisted of “specifically attending to the successive order of shapes, trying to identify the regularities between them, and checking explicit hypotheses made regarding these regularities” (p. 6).

Findings from our study serve as a starting point for future research by demonstrating not only that participants used strategies (including naming or labelling of stimuli) during the familiarisation phase of the visual statistical learning task, but also how they used these strategies to help them complete the familiarisation phase. Future research should investigate the use of strategies during the visual statistical learning task to understand *how* participants extract statistical regularities and whether the way in which participants extract statistical regularities affects visual statistical learning.

## Conclusion

This study extended the previous study by Cox et al. (2022), and it is only the second study to have tested the animate-monitoring hypothesis in visual statistical learning. In this study, we manipulated the animacy status of the stimuli in the unattended visual stream to determine whether this animacy manipulation would affect visual statistical learning of attended and unattended information. Replicating the findings in our previous study (Cox et al., 2022), we demonstrated that selective attention reliably modulates visual statistical learning. In addition, similar to our previous study, we did not find evidence of animacy effects on visual statistical learning of attended or of unattended information. There were no differences in visual statistical learning of attended and unattended information for the two animacy conditions. Although many studies have demonstrated effects of animacy in visual cognition (support for the animate-monitoring hypothesis), there are studies that have demonstrated no effects of animacy in visual cognition (no support for the animate-monitoring hypothesis). Future research should continue to investigate aspects of visual cognition where the animate-monitoring hypothesis is supported and also where it has not been supported as this would improve our understanding of how adaptive functions have evolved from ancestral environments, and shaped visual cognition.

## Notes

1 The factor “stimulus-repetition item” (i.e., the stimulus repetition presented in the familiarisation phase) was not included as a random intercept in the model for stimulus-repetition detection accuracy because the factor resulted in zero variance estimates. Including or excluding “stimulus-repetition item” as a random intercept in the model did not change the results or the conclusions from the model.

2 The random effects structure for this model indicated a singular fit, which means that the model is overfitted. Excluding the factor “visual stream” as a random slope in the model remediated the issue, such that, the model was no longer overfitted but the model without the random slope assumes independence among observations. Because “visual stream” is a within-subjects factor in our study design, we included this random slope in the model to ensure that the model is a maximal model that captures all the dependencies in the study sample (Barr et al., 2013; Heisig & Schaeffer, 2018). Including or excluding “visual stream” as a random slope in the model did not change the results or the conclusions from the model.

3 We are grateful to a reviewer for the suggestion about the experimental design that prompted the material in this paragraph.

## References

[bibr1-17470218231173883] Altman M. N. Khislavsky A. L. Coverdale M. E. Gilger J. W. (2016). Adaptive attention: How preference for animacy impacts change detection. Evolution and Human Behavior, 37(4), 303–314. 10.1016/j.evolhumbehav.2016.01.006

[bibr2-17470218231173883] Barr D. J. Levy R. Scheepers C. Tily H. J. (2013). Random effects structure for confirmatory hypothesis testing: Keep it maximal. Journal of Memory and Language, 68(3), 255–278. 10.1016/j.jml.2012.11.001 PMC3881361 24403724

[bibr3-17470218231173883] Bates D. Mächler M. Bolker B. Walker S. (2015). Fitting linear mixed-effects models using lme4. Journal of Statistical Software, 67(1), 1–48. 10.18637/jss.v067.i01

[bibr4-17470218231173883] Batterink L. J. Paller K. A. (2019). Statistical learning of speech regularities can occur outside the focus of attention. Cortex, 115, 56–71. 10.1016/j.cortex.2019.01.013 30771622 PMC6513683

[bibr5-17470218231173883] Bertels J. Destrebecqz A. Franco A. (2015). Interacting effects of instructions and presentation rate on visual statistical learning. Frontiers in Psychology, 6, Article 1806. 10.3389/fpsyg.2015.01806 PMC4663239 26648884

[bibr6-17470218231173883] Bertels J. Franco A. Destrebecqz A. (2012). How implicit is visual statistical learning? Journal of Experimental Psychology: Learning, Memory, and Cognition, 38(5), 1425–1431. 10.1037/a0027210 22329789

[bibr7-17470218231173883] Bonin P. Gelin M. Bugaiska A. (2014). Animates are better remembered than inanimates: Further evidence from word and picture stimuli. Memory & Cognition, 42(3), 370–382. 10.3758/s13421-013-0368-8 24078605

[bibr8-17470218231173883] Brodeur M. B. Dionne-Dostie E. Montreuil T. Lepage M. (2010). The Bank of Standardized Stimuli (BOSS), a new set of 480 normative photos of objects to be used as visual stimuli in cognitive research. PLOS ONE, 5(5), Article e10773. 10.1371/journal.pone.0010773 PMC2879426 20532245

[bibr9-17470218231173883] Bugaiska A. Grégoire L. Camblats A.-M. Gelin M. Méot A. Bonin P. (2019). Animacy and attentional processes: Evidence from the Stroop task. Quarterly Journal of Experimental Psychology, 72(4), 882–889. 10.1177/1747021818771514 29716460

[bibr10-17470218231173883] Calvillo D. P. Hawkins W. C. (2016). Animate objects are detected more frequently than inanimate objects in inattentional blindness tasks independently of threat. Journal of General Psychology, 143(2), 101–115. 10.1080/00221309.2016.1163249 27055078

[bibr11-17470218231173883] Calvillo D. P. Jackson R. E. (2014). Animacy, perceptual load, and inattentional blindness. Psychonomic Bulletin & Review, 21(3), 670–675. 10.3758/s13423-013-0543-8 24197657

[bibr12-17470218231173883] Cheesman J. Merikle P. M. (2001). Distinguishing conscious from unconscious perceptual processes. In Baun J. Koch C. Davis J. L. (Eds.), Visual attention and cortical circuits (pp. 519–540). MIT Press. 10.1037/h0080103 3502878

[bibr13-17470218231173883] Cosmides L. Tooby J. (2013). Evolutionary psychology: New perspectives on cognition and motivation. Annual Review of Psychology, 64, 201–229. 10.1146/annurev.psych.121208.131628 23282055

[bibr14-17470218231173883] Cox J. A. Aimola Davies A. M. (2022). Age differences in visual statistical learning: Investigating the effects of selective attention and stimulus category. Psychology and Aging, 37(6), 698–714. 10.1037/pag0000697 35878102

[bibr15-17470218231173883] Cox J. A. Cox T. W. Aimola Davies A. M. (2022). Are animates special? Exploring the effects of selective attention and animacy on visual statistical learning. Quarterly Journal of Experimental Psychology, 75(9), 1746–1762. 10.1177/17470218221074686 35001729

[bibr16-17470218231173883] Creel S. C. Newport E. L. Aslin R. N. (2004). Distant melodies: Statistical learning of nonadjacent dependencies in tone sequences. Journal of Experimental Psychology: Learning, Memory, and Cognition, 30(5), 1119–1130. 10.1037/0278-7393.30.5.1119 15355140

[bibr17-17470218231173883] Desimone R. Duncan J. (1995). Neural mechanisms of selective visual attention. Annual Review of Psychology, 18(1), 193–222. 10.1146/annurev.ne.18.030195.001205 7605061

[bibr18-17470218231173883] Dienes Z. (2004). Assumptions of subjective measures of unconscious mental states. Journal of Consciousness Studies, 11(9), 25–45.

[bibr19-17470218231173883] Duncan D. Theeuwes J. (2020). Statistical learning in the absence of explicit top-down attention. Cortex, 131, 54–65. 10.1016/j.cortex.2020.07.006 32801075

[bibr20-17470218231173883] Fox J. Weisberg S. (2019). An R companion to applied regression (3rd ed.). SAGE.

[bibr21-17470218231173883] Gelman R. Opfer J. E. (2002). Development of the animate-inanimate distinction. In Goswami U. (Ed.), Blackwell handbook of childhood cognitive development (pp. 151–166). Blackwell Publishing.

[bibr22-17470218231173883] Gelman R. Spelke E. S. (1981). The development of thoughts about animate and inanimate objects: Implications for research on social cognition. In Flavell J. H. Ross L. (Eds.), Social cognitive development: Frontiers and possible futures (pp. 43–66). Cambridge University Press.

[bibr23-17470218231173883] Guerrero G. Calvillo D. P. (2016). Animacy increases second target reporting in a rapid serial visual presentation task. Psychonomic Bulletin & Review, 23(6), 1832–1838. 10.3758/s13423-016-1040-7 27112561

[bibr24-17470218231173883] Hagen T. Espeseth T. Laeng B. (2018). Chasing animals with split attention: Are animals prioritized in visual tracking? I-Perception, 9(5), 1–35. 10.1177/2041669518795932 PMC6124190 30202509

[bibr25-17470218231173883] Hagen T. Laeng B. (2016). The change detection advantage for animals: An effect of ancestral priorities or progeny of experimental design? I-Perception, 7(3), 1–17. 10.1177/2041669516651366 PMC4934668 27433331

[bibr26-17470218231173883] Hagen T. Laeng B. (2017). Animals do not induce or reduce attentional blinking, but are reported more accurately in a rapid serial visual presentation task. I-Perception, 8(5), 1–25. 10.1177/2041669517735542 PMC5648101 29085619

[bibr27-17470218231173883] Heisig J. P. Schaeffer M. (2018). Why you should always include a random slope for the lower-level variable involved in a cross-level interaction. https://osf.io/mqu7z/

[bibr28-17470218231173883] Himberger K. D. (2021). Reconsidering visual statistical learning: Effects of attention, assessment, and associability [Doctoral dissertation]. John Hopkins University, John Hopkins University Sheridan Libraries. http://jhir.library.jhu.edu/handle/1774.2/64045

[bibr29-17470218231173883] Huang C. Theeuwes J. Donk M. (2021). Statistical learning affects the time courses of salience-driven and goal-driven selection. Journal of Experimental Psychology: Human Perception and Performance, 47(1), 121–133. 10.1037/xhp0000781 33119339

[bibr30-17470218231173883] JacksonR. E. CalvilloD. P. (2013). Evolutionary relevance facilitates visual information processing. Evolutionary Psychology, 11(5), 1011–1015. 10.1177/14747049130110050624184882

[bibr31-17470218231173883] Kim R. Seitz A. Feenstra H. Shams L. (2009). Testing assumptions of statistical learning: Is it long-term and implicit? Neuroscience Letters, 461(2), 145–149. 10.1016/j.neulet.2009.06.030 19539701

[bibr32-17470218231173883] Kirkham N. Z. Slemmer J. A. Johnson S. P. (2002). Visual statistical learning in infancy: Evidence for a domain general learning mechanism. Cognition, 83(2), B35–B42. 10.1016/s0010-0277(02)00004-5 11869728

[bibr33-17470218231173883] Klein R. P. Jennings K. D. (1979). Responses to social and inanimate stimuli in early infancy. The Journal of Genetic Psychology, 135(1), 3–9. 10.1080/00221325.1979.10533411 512642

[bibr34-17470218231173883] Kroll J. F. Potter M. C. (1984). Recognizing words, pictures, and concepts: A comparison of lexical, object, and reality decision. Journal of Verbal Learning and Verbal Behavior, 23(1), 39–66. 10.1016/s0022-5371(84)90499-7

[bibr35-17470218231173883] Lavie N. (2010). Attention, distraction, and cognitive control under load. Current Directions in Psychological Science, 19(3), 143–148. 10.1177/0963721410370295

[bibr36-17470218231173883] Lavie N. Beck D. N. Konstantinou N. (2014). Blinded by the load: Attention, awareness and the role of perceptual load. Philosophical Transactions of the Royal Society of London. Series B, Biological Sciences, 369(1641), Article 20130205. 10.1098/rstb.2013.0205 PMC3965161 24639578

[bibr37-17470218231173883] Lavie N. Hirst A. de Fockert J. W. Viding E. (2004). Load theory of selective attention and cognitive control. Journal of Experimental Psychology: General, 133(3), 339–354. 10.1037/0096-3445.133.3.339 15355143

[bibr38-17470218231173883] Legerstee M. (1992). A review of the animate-inanimate distinction in infancy: Implications for models of social and cognitive knowing. Early Development & Parenting, 1(2), 59–67. 10.1002/edp.2430010202

[bibr39-17470218231173883] Legerstee M. Pomerleau A. Malcuit G. Feider H. (1987). The development of infants’ responses to people and a doll: Implications for research in communication. Infant Behavior & Development, 10(1), 81–95. 10.1016/0163-6383(87)90008-7

[bibr40-17470218231173883] Lenth R. V. (2022). emmeans: Estimated marginal means, aka least-squares means [R package version 1.7.2]. https://CRAN.R-project.org/package=emmeans

[bibr41-17470218231173883] Li A.-S. Theeuwes J. (2020). Statistical regularities across trials bias attentional selection. Journal of Experimental Psychology: Human Performance and Performance, 46(8), 860–870. 10.1037/xhp0000753 32324032

[bibr42-17470218231173883] Milne A. E. Petkov C. I. Wilson B. (2018). Auditory and visual sequence learning in humans and monkeys using an artificial grammar learning paradigm. Neuroscience, 389, 104–117. 10.1016/j.neuroscience.2017.06.059 28687306 PMC6278909

[bibr43-17470218231173883] Moldwin T. Schwartz O. Sussman E. S. (2017). Statistical learning of melodic patterns influences the brain’s response to wrong notes. Journal of Cognitive Neuroscience, 29(12), 2114–2122. 10.1162/jocn_a_01181 28850296 PMC9248027

[bibr44-17470218231173883] Monroy C. D. Meyer M. Schröer L. Gerson S. A. Hunnius S. (2019). The infant motor system predicts actions based on visual statistical learning. NeuroImage, 185, 947–954. 10.1016/j.neuroimage.2017.12.016 29225063

[bibr45-17470218231173883] Morgan E. Fogel A. Nair A. Patel A. D. (2019). Statistical learning and Gestalt-like principles predict melodic expectations. Cognition, 189, 23–34. 10.1016/j.cognition.2018.12.015 30913527

[bibr46-17470218231173883] Musz E. Weber M. J. Thompson-Schill S. L. (2015). Visual statistical learning is not reliably modulated by selective attention to isolated events. Attention, Perception, & Psychophysics, 77(1), 78–96. 10.3758/s13414-014-0757-5 PMC4286474 25172196

[bibr47-17470218231173883] New J. Cosmides L. Tooby J. (2007). Category-specific attention for animals reflects ancestral priorities, not expertise. Proceedings of the National Academy of Sciences of the United States of America, 104(42), 16598–16603. 10.1073/pnas.0703913104 17909181 PMC2034212

[bibr48-17470218231173883] Otsuka S. Nishiyama M. Nakahara F. Kawaguchi J. (2013). Visual statistical learning based on the perceptual and semantic information of objects. Journal of Experimental Psychology: Learning, Memory, and Cognition, 39(1), 196–207. 10.1037/a0028645 22686848

[bibr49-17470218231173883] Rawal A. Tseng P. (2021). The effect of visual statistical learning in RSVP: Implicit learning or stream location artifact? Journal of Experimental Psychology: Learning, Memory, and Cognition, 47(8), 1246–1263. 10.1037/xlm0000988 33539166

[bibr50-17470218231173883] Saffran J. R. Aslin R. N. Newport E. L. (1996). Statistical learning by 8-month-old infants. Science, 274(5294), 1926–1928. 10.1126/science.274.5294.1926 8943209

[bibr51-17470218231173883] Saffran J. R. Newport E. L. Aslin R. N. Tunick R. A. Barrueco S. (1997). Incidental language learning: Listening (and learning) out of the corner of your ear. Psychological Science, 8(2), 101–105. 10.1111/j.1467-9280.1997.tb00690.x

[bibr52-17470218231173883] Sáringer S. Fehér Á. Sáry G. Kaposvári P. (2022). Online measurement of learning temporal statistical structure in categorization tasks. Memory and Cognition. 50, 1530–1545. 10.3758/s13421-022-01302-5 35377057 PMC9508059

[bibr53-17470218231173883] Searle S. R. Speed F. M. Milliken G. A. (1980). Population marginal means in the linear model: An alternative to least squares means. The American Statistician, 34(4), 216–221. 10.1080/00031305.1980.10483031

[bibr54-17470218231173883] Sherman B. E. Graves K. N. Turk-Browne N. B. (2020). The prevalence and importance of statistical learning in human cognition and behaviour. Current Opinion in Behavioral Sciences, 32, 15–20. 10.1016/j.cobeha.2020.01.015 32258249 PMC7108790

[bibr55-17470218231173883] Snodgrass J. G. Vanderwart M. (1980). A standardized set of 260 pictures: Norms for name agreement, image agreement, familiarity, and visual complexity. Journal of Experimental Psychology: Human Learning and Memory, 6(2), 174–215. 10.1037/0278-7393.6.2.174 7373248

[bibr56-17470218231173883] Thomas P. M. J. Jackson Margaret C. Raymond J. E. (2014). A threatening face in the crowd: Effects of emotional singletons on visual working memory. Journal of Experimental Psychology: Human Perception and Performance, 40(1), 253–263. 10.1037/a003970 23957307

[bibr57-17470218231173883] Turk-Browne N. B. Jungé J. A. Scholl B. J. (2005). The automaticity of visual statistical learning. Journal of Experimental Psychology: General, 134(4), 552–564. 10.1037/0096-3445.134.4.552 16316291

[bibr58-17470218231173883] VanVoorhis C. R. W. Morgan B. L. (2007). Understanding power and rules of thumb for determining sample sizes. Tutorials in Quantitative Methods for Psychology, 3(2), 43–50. 10.20982/TQMP.03.2.P043

[bibr59-17470218231173883] Wierzchoń M. Asanowicz D. Paulewicz B. Cleeremans A. (2012). Subjective measures of consciousness in artificial grammar learning task. Consciousness and Cognition, 21(3), 1141–1153. 10.1016/j.concog.2012.05.012 22728143

